# Spinal Reflex Control of Arterial Blood Pressure: The Role of TRP Channels and Their Endogenous Eicosanoid Modulators

**DOI:** 10.3389/fphys.2022.838175

**Published:** 2022-02-23

**Authors:** Zeljka Minic, Donal S. O’Leary, Christian A. Reynolds

**Affiliations:** ^1^Department of Emergency Medicine Wayne State University School of Medicine, Detroit, MI, United States; ^2^Department of Biotechnology, University of Rijeka, Rijeka, Croatia; ^3^Department of Physiology, Wayne State University School of Medicine, Detroit, MI, United States

**Keywords:** blood pressure (BP), spinal cord injury, eicosanoids in physiological and pathological processes, transient receptor potential (TRP) channel, visceral reflexes, sympathetic nerve activity (SNA)

## Abstract

The spinal cord is an important integrative center for blood pressure control. Spinal sensory fibers send projections to sympathetic preganglionic neurons of the thoracic spinal cord and drive sympathetically-mediated increases in blood pressure. While these reflexes responses occur in able-bodied individuals, they are exaggerated following interruption of descending control – such as occurs following spinal cord injury. Similar reflex control of blood pressure may exist in disease states, other than spinal cord injury, where there is altered input to sympathetic preganglionic neurons. This review primarily focuses on mechanisms wherein visceral afferent information traveling *via* spinal nerves influences sympathetic nerve activity and blood pressure. There is an abundance of evidence for the widespread presence of this spinal reflex arch originating from virtually every visceral organ and thus having a substantial role in blood pressure control. Additionally, this review highlights specific endogenous eicosanoid species, which modulate the activity of afferent fibers involved in this reflex, through their interactions with transient receptor potential (TRP) cation channels.

## Introduction

Physiological mechanisms of blood pressure control involve complex interactions between neural and humoral factors, which work hand-in-hand to ensure optimal delivery of nutrient rich blood to all the cells of the body. In regulating blood pressure, particularly important are the effects of the sympathetic nervous system. Sympathetic postganglionic neurons innervate blood vessels to regulate vascular smooth muscle tone and peripheral resistance. The rostral ventrolateral medulla is arguably one of the most critical cites for establishing baseline blood pressure as it sends tonic impulses to sympathetic preganglionic neurons located within the intermediolateral cell column of the thoracic spinal cord. Although the spinal cord is often described as a mere relay station between the brainstem and the periphery, clinical and experimental evidence highlights the important role of intraspinal reflex circuits in blood pressure regulation.

In this review we summarize available evidence that suggests intraspinal reflex circuits can directly and independently orchestrate profound reflex-mediated changes in blood pressure, especially when descending inhibitory pathways are interrupted, as in patients with spinal cord injuries. We primarily focus on mechanisms wherein visceral afferent information traveling *via* spinal nerves influences sympathetic nerve activity and blood pressure. Special emphasis is placed on descending inhibitory control of sympathetic preganglionic neurons and how interruption of this inhibition, as occurs following spinal cord injury, leads to aberrant spinal reflex control of blood pressure. Additionally, we propose that the spinal sensory input may contribute to tonic control of blood pressure in able bodied individuals. Finally, we highlight endogenous modulators of this neurocircuitry and their potential roles in regulation of blood pressure in healthy and diseased states.

## Viscero-Sympathetic Reflexes

By the early 1800’s it had been established that the spinal cord served as a relay station for passage but not integration of neuronal information. In 1833 Marshall Hall first concluded that the spinal cord is capable of orchestrating meaningful physiological responses to visceral stimulation (Hall, 1833; Pearce, 1997). These responses are mediated *via* a spinal reflex arc – independent of central processing within the brain. The effect of visceral stimulation on systemic blood pressure was first empirically demonstrated in seminal works by Sir Charles Sherrington. He showed that distension of hollow visceral organs such as the urethra, bile duct or rectum in spinalized or decerebrate animals led to moderate increases in blood pressure, which were associated with a series of involuntary motor responses (Sherrington and Laslett, 1903). Comparable pressor/motor responses were recapitulated using similar visceral stimuli in other animal preparations (Mackenzie, 1906; Miller, 1924; Miller and Waud, 1925; Lewis, 1939; Downman and McSwiney, 1946). Miller and colleagues reported that pinching of the small intestine, pulling the mesentery, or faradizing mesenteric nerves produced contraction of leg and belly muscles with a rise in blood pressure in decapitate cats (Miller, 1924; Miller and Waud, 1925). Similarly, in decapitate cats, mechanical stimulation of the pancreas produced increase in blood pressure and abdominal contractions (Lewis, 1939). Finally, Downman and McSwiney (1946) reported pressor responses to mechanical stimulation of the jejunal mesentery, ileum and scrotum in spinalized cats.

Although the mechanisms contributing to the blood pressure increases were not elucidated in these early works, some authors reported that the pressor responses to visceral stimulations were associated with pale discoloration of the visceral organ where the stimuli originated and extended into surrounding tissues (Downman and McSwiney, 1946). Furthermore, it was noted that the natural color of the organ was restored as the blood pressure returned to baseline, suggesting that sympathetic vasoconstriction was responsible for the observed changes in the organ’s color. This early experimental evidence supported the idea that visceral sensory information can directly influence cardiovascular function *via* activation of a viscero-sympathetic reflex (Häbler et al., 1992). This reflex has also been referred to as viscero-vascular reflex or viscero-visceral reflex (Taylor, 1968; Conci et al., 1986).

## Neural Circuitry of the Viscero-Sympathetic Reflex

**FIGURE 1:**
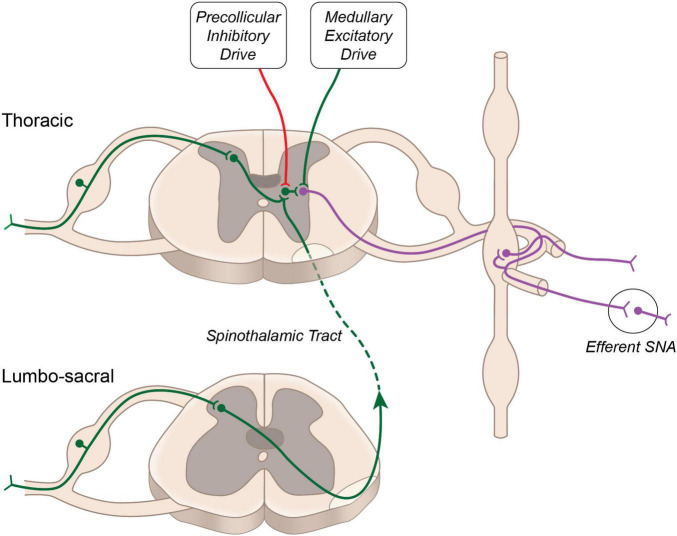
Neural circuitry regulating sympathetic nerve activity. Major excitatory drive to sympathetic preganglionic neurons arises from the rostral ventrolateral medulla. Additional excitatory input from propriospinal projecting neurons is buffered by descending inhibitory drive arising above the level of the superior colliculus.

Sensory visceral stimuli produced by physiological and pathophysiological changes (thermal, chemical, or mechanical) within and surrounding visceral organs, are relayed to the central nervous system *via* cranial (e.g., vagal) and spinal pathways. Stimulation of cranial afferent nerve fibers is generally associated with cardiovascular depressor responses, while spinal afferent stimulation generally evokes pressor responses (Tjen-A-Looi et al., 1997; Longhurst, 2020). First order neurons carrying sensory information from the viscera into the spinal cord are composed of thinly myelinated Aδ and unmyelinated C fibers. These fibers run alongside efferent sympathetic fibers and have cell bodies in the dorsal root ganglion. Central projections of these neurons enter the spinal cord through the dorsal horn and form synapses with second order neurons. In general, C-fibers project to lamina II (substantia gelatinosa) and lamina III (nucleus proprius) of the dorsal horn, while Aδ fibers terminate primarily within lamina I and lamina V (Steeds, 2009). Within the dorsal horn sensory information is directed to: (i) interneurons, which terminate within the same spinal segment, (ii) propriospinal neurons, which terminate within a different spinal segment, and (iii) projecting neurons, which terminate within supraspinal structures. Activation of sympathetic preganglionic neurons occurs either *via* second order interneurons, second order propriospinal neurons or second order spinothalamic projecting neurons, which have extensive axon collaterals within the thoracic spinal cord (Browne et al., 2020) (Figure 1). Additional interneurons (third order) terminating within the intermediolateral cell column of the spinal cord are likely critical for sensory-sympathetic coupling, as second order neurons (arising from the dorsal horn) rarely make direct connections with cell bodies of sympathetic preganglionic neurons (Schramm, 2006).

Activation of this reflex circuitry triggers the viscero-sympathetic reflex, which increases efferent sympathetic nerve activity and systemic blood pressure. In addition to activating sympathetic preganglionic neurons at the level of the spinal cord, the second order spinothalamic projecting neurons also send collateral projections to the several brainstem nuclei and higher brain centers such as hypothalamus, thalamus and cortex. These central structures may modulate the viscero-sympathetic reflex through descending projections to the spinal cord. Spinal enkephalinergic interneurons are activated by descending projection from the Raphe nucleus to release enkephalin, an endogenous opioid that inhibits second order neurons and thus attenuates viscero-sympathetic reflex activation. Additional enkephalinergic interneurons have been found to terminate on sympathetic preganglionic neurons (Llewellyn-Smith and Weaver, 2004), and their number was increased in the chronic stage following spinal cord injury (Hou et al., 2008), suggesting potential for modulation of the viscero-sympathetic reflex through this endogenous opioid system.

However, the purpose of this review is to highlight and remind the reader that the critical integrative center for this reflex is located at the level of the spinal cord and it is *via* this and similar neuronal pathways that the spinal cord likely contributes to blood pressure regulation in healthy and diseased states.

The viscero-sympathetic reflex circuitry is similar in composition to the spinal motor reflex circuits that drive muscle contraction in response to muscle stretch or cutaneous stimulation, and like spinal motor reflexes, the viscero-sympathetic reflex is exaggerated by spinal cord injury and decerebration (Sherrington and Laslett, 1903; Miller, 1924; Miller and Waud, 1925; Lewis, 1939; Downman and McSwiney, 1946). It is well appreciated that descending corticothalamic projections exhibit tonic inhibitory drive to spinal motoneurons (Jankowska et al., 1976; Triggs et al., 1992). Similarly, we propose that descending inhibitory projections arising from above the level of the brainstem modulate viscero-sympathetic reflex activation. Such descending inhibitory control likely arises from cortical or hypothalamic centers and accounts for the exaggerated sympathetic responses to visceral stimulation following decerebration at the level of the midbrain which thereby transects these inhibitory pathways. As others before us, we propose that viscero-sympathetic and other spinal reflexes evolved in early invertebrates and were suppressed by later-evolving higher brain structures through a process of phylogenetic refinement (Spittler et al., 2000). Therefore, the “older” visceral control of the cardiovascular system is set free when the cord is uncoupled from the “younger” inhibitory input of higher brain structures.

Evidence exists for cortical modulation of sympathetic nerve activity in response to emotional or psychological stress (Berntson et al., 1998), and cortical stimulation or lesioning of cortical structures can produce changes in sympathetic nerve activity and cardiovascular responses (Hoff et al., 1963; Cechetto and Saper, 1990). Another central structure that likely exerts inhibitory control over sympathetic preganglionic neurons is the periaqueductal gray, and deep brain stimulation of this region dramatically reduces systemic blood pressure in patients with refractory hypertension associated with neuropathic pain (O’Callaghan et al., 1979; O’Callaghan et al., 2014). These effects are likely mediated through central projections to hypothalamic and lower brainstem centers where basic cardiovascular reflexes are integrated (Cechetto and Chen, 1992; Sequeira et al., 2000). Although harder to demonstrate, it remains possible that projections from higher brain structures may modulate sympathetic preganglionic neurons more directly, through descending projections to the thoracic spinal cord. To this end, axons of the corticospinal tract can form close appositions with sympathetic preganglionic neurons (Pan et al., 2005) and stimulation of corticospinal tract can directly influence sympathetic nerve activity (Pan et al., 2007).

## Viscero-Sympathetic Reflexes in Humans

The clinical importance of viscero-sympathetic reflexes is most easily appreciated in individuals with cervical or high thoracic spinal cord injury. In these patients, otherwise benign distension of the bladder or colon triggers activation of the viscero-sympathetic reflex, which produces vigorous pressor responses, a phenomenon commonly referred to as *autonomic dysreflexia*. Guttmann and Whitteridge (1947) demonstrated that distension of the bladder in a human with high thoracic spinal cord injury evokes episodic increases in blood pressure, which are associated with baroreflex-mediated decreases in heart rate and vasodilation of vascular beds innervated by the sympathetic neurons from above the level of the injury. If the spinal cord injury is located above the level of the cardiac sympathetic output (i.e., higher cervical injuries), the reflex-mediated bradycardia is absent and a tachycardiac response is observed (Eldahan and Rabchevsky, 2018). Similar cardiovascular responses can be produced by cutaneous stimulation below the level of the spinal cord injury through activation of a somato-sympathetic reflex (Brown et al., 2007, 2009; Macefield et al., 2012). In addition to cardiovascular responses, activation of viscero-sympathetic reflex can result in throbbing headache, nausea, irritability, and sometimes even convulsion, cerebral hemorrhage or death (Eldahan and Rabchevsky, 2018). As it is the case in the animal studies, the reflex mediated changes in blood pressure in humans are sympathetically mediated as they are abolished by ganglionic blockade (Kurnick, 1956; Corbett et al., 1971; Fagius and Karhuvaara, 1989; Osborn et al., 1990).

Spinal cord injury leaves sympathetic preganglionic neurons devoid of descending inhibitory control leaving excitation of the viscero-sympathetic reflex arc by sensory afferents unopposed (Llewellyn-Smith, 2002; Eldahan and Rabchevsky, 2018). Although sympathetic preganglionic neurons that control blood pressure are located between T1 and L2 spinal segments, it is generally accepted that individuals with injuries at T6 and higher may experience autonomic dysreflexia. Furthermore, the likelihood of autonomic dysreflexia is increased as the level of the spinal cord injury is increased.

Importantly, cardiovascular responses to visceral stimulation are observed in the individuals with an intact neuraxis, but responses are especially vigorous in individuals with interruption of descending inhibitory control (Ness and Gebhart, 1988). In fact, it is not uncommon to observe massive pressor responses to visceral stimulation in brain dead organ donors, despite a complete lack of brainstem function (Conci et al., 1986). These data suggest that the spinal cord, especially when it is devoid of the descending influence from higher brain centers, is capable of controlling blood pressure through intraspinal reflex circuits.

## Visceral Afferent Fibers

Visceral sensation is complex and less well understood than somato-sensation. One of the most fundamental differences is in the types of stimuli which drive visceral sensation. In general, visceral afferent fibers are strongly activated by diffuse stimuli such as pressure as occurring during distension of hollow visceral organs, changes in pH, heat, or inflammation. Even though the majority of viscero-sensory information does not reach our consciousness, there is ample evidence that visceral organs are highly responsive to various stimuli and are in continuous communication with spinal and supraspinal structures. Some examples of continuous sensory input supplied by the viscera include hepatic osmoreceptors, and mechanoreceptors of the stomach, bladder and colon involved in detecting fullness or facilitating peristalsis.

Unlike the upper visceral organs (e.g., esophagus and stomach), the lower visceral organs are arguably less innervated by vagal afferent fibers and are densely innervated by thoracolumbar and/or sacral (pelvic) afferent fibers, whose cells bodies are located within dorsal root ganglia. The nerve bundles of these spinal sensory neurons are composed of thinly myelinated Aδ and unmyelinated C fibers (Sengupta and Gebhart, 1994a,b). These fibers are polymodal, mostly responding to mechanical, chemical, and thermal stimuli (Blumberg et al., 1983; Sengupta, 2009).

Visceral mechano sensation has been extensively studied and mechanosensitive afferent fibers arising from the viscera are important drivers of sympathetic reflexes (Sengupta and Gebhart, 1994a).

Low threshold mechanosensitive afferent fibers innervate mucosal membranes and muscle layers of the visceral organs and are tonically active (Grundy et al., 2019). They are involved in the detection of luminal content, exhibit a wide dynamic range, and respond with linear increases in firing in response to increased distension pressure (Jänig, 1996; de Groat and Yoshimura, 2009). Under physiological conditions, they are responsible for detection of fecal matter consistency and composition, bladder fullness and for coordination of autonomic emptying reflexes. They also contribute to nociception at high levels of distension.

High threshold mechanosensitive afferent fibers innervate blood vessels in the mesentery and mucosal membranes and muscle layers of visceral organs. These fibers are involved in detecting urgency, changes in mesenteric arterial pressure and mechanically induced pain.

Silent mechanosensitive afferent fibers are mostly involved in signaling pathological visceral changes (Feng and Gebhart, 2011). These fibers become mechanosensitive in response to chemical/inflammatory stimuli, and generally exhibit firing properties like those of high threshold fibers.

It is well established that mechanosensitive Aδ and C-fibers drive sympathetic responses to mechanical stimulation of the viscera (Sengupta and Gebhart, 1994a). However, it remains unclear if any of the above-mentioned subpopulations of mechanosensitive afferent fibers predominate in activating viscero-sympathetic reflex.

The above described functional organization for processing of afferent information exists within most spinal segments and highlights potential of intraspinal circuitry to influence spinal visceral reflexes originating from various spinal segments.

## Additional Spinal Visceral Reflexes

As described above, the viscero-sympathetic reflex, similar to spinal motor reflexes, is integrated at the level of the spinal cord. Various visceral reflex arcs are contained within the spinal cord and are involved in normal gastrointestinal and urogenital physiology. As indicated below, activation of most spinal visceral reflexes is preserved following upper thoracic spinal cord injury and is associated with increases in systemic blood pressure.

**Micturition reflex:** During the storage phase of the micturition reflex, pelvic sensory afferents send slow impulses into the spinal cord, which drive involuntary contraction of the internal urethral sphincter and relaxation of the detrusor muscle, both of which are needed to facilitate urine retention and are mediated *via* sympathetic efferent fibers. Importantly, bladder filling is associated with a progressive increase in activity of other regional sympathetic efferent fibers and in systemic blood pressure (Taylor, 1965; Fagius and Karhuvaara, 1989). Furthermore, splanchnic sympathectomy abolishes pressor responses to bladder filling suggesting the neurogenic origin of the blood pressure increase (Mukherjee, 1957). Neither baroreceptor denervation nor high thoracic spinal cord injury interfere with bladder urine retention or associated pressor responses, which firmly indicate that integration occurs at the level of the spinal cord (Stjernberg et al., 1986; Krum et al., 1992; Cheng et al., 1995; Tai et al., 2006). Conversely, the voiding phase of the micturition reflex is voluntary, modulated mostly by the pontine micturition center, and lost following spinal cord injury.

**Defecation reflex:** Much like in the case of the bladder, distension of the lower gastrointestinal tract is associated with increases in sympathetic nerve activity and blood pressure in healthy people (Ness and Gebhart, 1988; Rossi et al., 1998; Miura et al., 1999; Timar-Peregrin et al., 2001). These pressor responses are related to activation of the spinal defecation reflex *via* distension of the rectum, which sends afferent information into lumbosacral spinal cord to activate efferent pathways that induce peristaltic contractions of the rectum and relaxation of the internal anal sphincter. These reflex functions are also preserved following thoracic spinal transection and are controlled at the level of the spinal cord.

**Osmoregulatory reflex:** Activation of peripheral osmoreceptors by hypo-osmotic stimuli (e.g., ingestion of water) results in reflex increases in sympathetic nerve activity and blood pressure (Scott et al., 2001; Tank et al., 2003; Jordan, 2005). This reflex is retained following spinal cord injury (Tank et al., 2003) and involves osmosensitive spinal afferent fibers that innervate hepatic tissues surrounding the portal vein (Lechner et al., 2011).

**Ejaculation reflex:** The ejaculation reflex is a spinal reflex that is initiated by the relay of afferent input from the perineal branch of the pudendal nerve to preganglionic sympathetic neurons of the thoracolumbar spinal cord (T10-L2). Sympathetic fibers innervating the bladder neck, prostate, seminal vesicles, and vas deferens are activated and drive the emission phase of ejaculation (Revenig et al., 2014). Activation of the ejaculation reflex is associated with transient increases in blood pressure in able-bodied men, and the pressor responses associated with ejaculation are exaggerated in men with upper thoracic or cervical spinal cord injuries (Revenig et al., 2014).

**Renorenal reflex:** The role of renal afferent fibers in regulating systemic blood pressure and cardiovascular disease has been extensively studied. Activation of renal afferent fibers results in reflex increases in efferent sympathetic nerve activity and systemic blood pressure and renal afferent denervation was demonstrated to prevent development of hypertension in various animal models (Wyss et al., 1986; Oparil et al., 1987; Campese and Kogosov, 1995; Ciriello and de Oliveira, 2002; Schlaich et al., 2012). Most of the literature published on the topic suggests that the antihypertensive effects of renal denervation result from removal of the afferent input into central (e.g., brainstem and hypothalamic) structures, which indirectly reduces efferent sympathetic nerve activity by reducing (descending) pacemaker input to sympathetic preganglionic neurons. However, anatomical studies suggest that renal afferents may also directly drive sympathetic preganglionic neurons *via* spinal mechanisms (Kuo et al., 1983; Simon and Schramm, 1984; Ciriello and de Oliveira, 2002). Consistent with this notion, activation of excitatory renorenal reflex *via* chemical stimuli or renal ischemia increases sympathetic nerve activity and blood pressure in spinal cord transected animals as it does in animals with an intact neuroaxis (Recordati et al., 1982; Kopp et al., 1984). One publication notes that spinal transection attenuates renorenal reflex activation, however, those experiments were performed 18 h following spinal cord transection (Kopp et al., 1985), at which point spinal shock and areflexia persist (Meshkinpour et al., 1985; Holmes et al., 1998; Ditunno et al., 2004). Thus, it is tempting to speculate that the antihypertensive effects of renal denervation result, at least in part, from attenuation of spinal reflex reactivity.

## Transient Receptor Potential Family of Ion Channels Involved in Activation of Sensory Afferents

Among the ion channels involved in activation of visceral afferent neurons are the non-selective cation channel members of the transient receptor potential (TRP) family. To date, 28 mammalian TRP channels have been identified (reviewed in Venkatachalam and Montell (2007); Mickle et al. (2015)), which are classified into six sub-families: vanilloid (TRPV), ankyrin (TRPA), melastatin (TRPM), canonical (TRPC), polycystin (TRPP), and mucolipin (TRPML). While TRPC and TRPM channels expressed within the kidney and on vascular smooth muscle cells have been implicated in blood pressure regulation (Dietrich et al., 2005, 2010; Zholos et al., 2011; Shin et al., 2019), the neuronally-expressed TRPV, TRPA, and TRPM family members are most directly implicated in the activation of spinal sensory afferents and spinal reflexes. Although TRP channels closely resemble voltage-gated ion channels in their structure, they generally are not voltage sensitive (Cao, 2020). Neuronally-expressed TRP channels are almost exclusively non-selective cation channels that allow for movement of both monovalent and divalent cations (e.g., Na^+^, and Ca^2+^) across the plasma membrane. TRP channels are activated by a wide range of physiological and pathophysiological signals and respond to both chemical ligands and physical stimuli. In addition, many of the mammalian TRP channels are thermo-sensitive and can be directly activated by cold or hot temperatures (Cao, 2020).

The TRPV subfamily was the first of the TRP channel families to be implicated in nociception. TRPV channels where so named because of their activation by vanilloid compounds, such as capsaicin, the spicy compound in chili peppers. TRPV1 is expressed in approximately 50% of C fibers and in a slightly smaller percentage of Aδ fibers (Kobayashi et al., 2005). Thus, it is not surprising that TRPV1 channels located on visceral afferent fibers can contribute to visceral-sympathetic reflex activation. TPRV1 expressing renal afferent fibers are key contributors to the development of hypertension and potent activators of preganglionic sympathetic neurons located in the thoracic spinal cord. Recent studies have confirmed that selective ablation of TRPV1 expressing renal afferent nerves *via* periaxonal application of high-dose capsaicin attenuates deoxycorticosterone acetate (DOCA) salt-sensitive hypertension (Banek et al., 2016).

TRPV4 channels appear to be critical for hepatic afferent responsivity to hypo-osmotic stimuli (Lechner et al., 2011). Importantly, the cardiovascular pressor responses associated with activation of hepatic osmosensitive afferents are abolished in the TRPV4^–/–^ mice, while TRPV1^–/–^ mice exhibit normal hepatic osmoreceptor activation and retain all associated pressor responses to hypo-osmotic stimuli.

In humans with spinal cord injury, intravesical administration of the TRPV1 agonist, capsaicin, elicits profound cardiovascular pressor responses (Igawa et al., 2003). Similarly, in the spinal transected rat, we observed dose-dependent pressor responses to enema administration of TRPV1 agonists, which are exaggerated when compared to animals with an intact neuroaxis (Minic et al., 2021). Furthermore, consistent with the findings indicating that TRPV1 agonists sensitize mechanosensitive fibers and lower their threshold of activation to mechanical stretch (Wu et al., 2001; Numazaki and Tominaga, 2004; Avelino and Cruz, 2006; McGaraughty et al., 2006; Daly et al., 2007), we also observed that administration of TPRV1 agonists sensitizes spinal cord transected animals to viscero-sympathetic reflex activation *via* colorectal distension (Minic et al., 2021). Taken altogether, these data suggest that channels play an important role in driving pressor responses to visceral stimuli both in humans and rodents.

## Eicosanoid-Transient Receptor Potential Interactions and Spinal Afferent Hyperexcitability

Irritants derived from pepper, mint, and mustard plants were some of the first identified agonists of TRP channels and have served as valuable pharmacological tools for the study of nociception. In recent decades multiple endogenous activators of TRP channels have been identified. TRP channels can be directly activated (e.g., peptide toxins, capsaicin and various bioactive lipids) or can be indirectly activated (e.g., bradykinin, ATP, and nerve growth factor) *via* second messenger–signaling pathways (reviewed in Julius (2013)). By far, the largest class of endogenous TRP ligands are bioactive lipid molecules such as endocannabinoids and eicosanoids (reviewed in Bang et al. (2010); Shapiro et al. (2016); Muller et al. (2018); Storozhuk and Zholos (2018)), which can sensitize spinal afferent neurons by lowering their threshold of activation.

**FIGURE 2:**
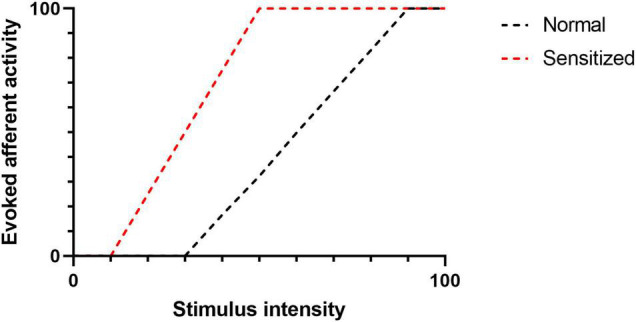
The figure illustrates evoked afferent nerve activity as a factor of stimulus intensity. Following sensitization (e.g., TRP-Eicosanoid binding), the activation threshold (intersection of a curve with the X -axis) is reduced. Similarly, the magnitude of the afferent response to a given stimulus is increased.

Eicosanoids are bioactive metabolites derived from polyunsaturated fatty acids (PUFAs) and generated *via* three major enzyme systems: the cyclooxygenase (COX), lipoxygenase (LOX) and cytochrome P450 (CYP) pathways. Eicosanoid molecules play a critical role in inflammatory signaling (Dennis and Norris, 2015; Maddipati, 2020), and their pro-algesic or analgesic effects are partially mediated through interactions with TRP channels. Early studies of the COX-derived eicosanoid, prostaglandin E_2_ (PGE_2_), clearly indicate that binding of PGE_2_ to its G protein coupled receptors (EP1-4) on the surface of TRP channel expressing neurons promotes hyperalgesia by driving phosphorylation of TRP channels thereby increasing excitability to mechanical, thermal and chemical stimuli (reviewed in Pethő and Reeh (2012)). However, in recent years it has become apparent that multiple eicosanoid species can act as endogenous TRP channel ligands and elicit dose-dependent afferent hyperexcitability *via* direct interactions (Table 1). Such interactions can (i) lower the activation threshold of the afferent fiber and/or (ii) increase the magnitude of the afferent response to a given stimulus (Figure 2).

**TABLE 1:** Eicosanoid metabolites activating TRP Channels.

Precursor PUFA	Eicosanoid lipid mediator	TPR channel interaction(s)	Reference(s)
Linoleic acid	● 13-hydroxy-9Z,11E-octadecadienoic acid (13-HODE)	TRPA1, TRPV1, TRPV2, agonist	Patwardhan et al., 2009, 2010; De Petrocellis et al., 2012
	● 13-oxo-9Z,11E-octadecadienoic acid (13-oxo-HODE)	TRPV1 agonist	Patwardhan et al., 2009, 2010
	● 9-hydroxy-10E,12Z-octadecadienoic acid (9-HODE)	TRPA1, TRPV1 agonist	Patwardhan et al., 2009, 2010; De Petrocellis et al., 2012
	● 9-ox0-10E,12Z-octadecadienoic acid (9-oxo-HODE)	TRPV1 agonist	Patwardhan et al., 2009, 2010
	● 9,10-dihydroxy-12Z-octadecenoic acid (9,10-DiHOME)	TRPA1, TRPV1 agonist	Eskander et al., 2015; Green et al., 2016; Sisignano et al., 2016
	● 9,10-epoxy-12Z-octadecenoic acid (9(10)EpOME)	TRPA1, TRPV1 agonist	Eskander et al., 2015; Green et al., 2016; Sisignano et al., 2016
	● 12,13-dihydroxy-9Z-octadecenoic acid (12,13-DiHOME)	TRPA1, TRPV1 agonist	Eskander et al., 2015; Green et al., 2016; Sisignano et al., 2016; Zimmer et al., 2018
	● 12(13)epoxy-9Z-octadecenoic acid (12(13)EpOME)	TRPA1, TRPV1 agonist	Eskander et al., 2015; Green et al., 2016; Sisignano et al., 2016
Arachidonic acid	● 5,6-epoxy-8Z,11Z,14Z-eicosatrienoic acid (5,6-EET)	TRPA1, TRPV4 agonist	Watanabe et al., 2003; Sisignano et al., 2012
	● 8,9-epoxy-5Z,11Z,14Z-eicosatrienoic acid (8,9-EET)	TRPA1, TRPV4 agonist	Watanabe et al., 2003
	● Prostaglandin A1 (PGA1)	TPPA1 agonist	Materazzi et al., 2008
	● Prostaglandin A2 (PGA2)	TPPA1 agonist	Materazzi et al., 2008
	● 8-iso Prostaglandin A2 (8-iso-PGA2)	TRPA1 agonist	Hwang et al., 2000
	● 12S-hydroperoxy-5Z,8Z,10E,14Z-eicosatetraenoic acid (12-HpETE)	TRPV1 agonist	Hwang et al., 2000
	● 15S-hydroperoxy-5Z,8Z,11Z,13E-eicosatetraenoic acid (15-HpETE)	TRPV1 agonist	Hwang et al., 2000
	● 5S-hydroxy-6E,8Z,11Z,14Z-eicosatetraenoic acid (5-HETE)	TRPV1 agonist	Hwang et al., 2000
	● Leukotriene B4 (LTB4)	TRPV1 agonist	Hwang et al., 2000

Interestingly, many of the eicosanoids that promote afferent hyperexcitability through interactions with TRP channels are derived from ω-6 PUFAs, which are abundant in Western diets. This has led us and others to hypothesize that dietary PUFA content may directly influence the excitability of TPR expressing C and Aδ fibers. Evidence to support this hypothesis was recently published by the laboratory of K.M. Hargeaves (Boyd et al., 2021). Specifically, the authors observed that mice maintained on a diet with high ω-6 PUFA content displayed nociceptive hypersensitivities and C fiber hyper-responsivity when compared to animals maintained on a diet with low ω-6 PUFA content. It is therefore tempting to speculate that dietary PUFA content may indirectly modulate spinal sympathetic reflexes by altering spinal afferent excitability.

## Conclusion and Future Perspectives

Spinal sympathetic reflex arcs are important drivers of normal physiological responses and the spinal cord itself is an integrative center for maintenance of arterial blood pressure. Spinal afferent fibers arising from the viscera robustly activate sympathetic preganglionic neurons *via* intraspinal circuitry. However, it is important to highlight that similar sympathetic responses can be generated by spinal afferent information arising from somatic afferents (e.g., muscle metaboreflex or exercise pressor reflex) (Victor et al., 1989; Toney and Mifflin, 2000; Murphy et al., 2011; Spranger et al., 2013). As in the case of the visceral afferents, somatic afferents involved in activation of pressor reflexes in response to muscle contraction are composed of thinly myelinated Aδ and unmyelinated C fibers (Coote et al., 1971). While it is often cited that central structures are required for activation of the exercise pressor reflex, this is based on a single observation, which demonstrated that acute cervical spinal cord transection eliminated the reflex in cats (Mitchell et al., 1983). However, in these studies the authors tested the reflex in the hours immediately after the spinal cord injury, during a period when spinal shock and complete areflexia are common (Ditunno et al., 2004). This leaves the possibility that somato-sympathetic reflexes, including the exercise pressor reflex, are integrated at the level of the spinal cord and if so, these reflexes may be activated in individuals with spinal cord lesions.

We have briefly highlighted the potential role of eicosanoid-TRP interactions in modulating the afferent limb of the viscero-sympathetic reflex circuitry. However, many mechanistic issues remain to be resolved and *in vivo* electrophysiology recording will be an important tool for understanding the role of eicosanoids in altering spinal afferent excitability. Furthermore, additional studies into the role of dietary PUFA content and eicosanoid-TRP interactions in regulating baseline sympathetic nerve activity are needed.

It is notable that inflammatory conditions involving the viscera, such as inflammatory bowel disease, lead to the development of visceral afferent hyperexcitability (Akbar et al., 2009; Kanazawa et al., 2011). Interestingly, multiple studies have identified an increased incidence of hypertension in patients with inflammatory bowel disease (Ha et al., 2009; Osterman et al., 2011; Kristensen et al., 2013) and it is tempting to speculate that spinal afferent hyperexcitability in these patients contributes to hypertension *via* chronic activation of the viscero-sympathetic reflex circuitry. Consistent with this notion, a large Danish cohort study found the risk for developing hypertension was significantly reduced among patients undergoing surgical colectomy when compared to patients undergoing other surgical procedures (Jensen et al., 2015). Future studies using animal models are required to clarify if and how spinal afferent fibers arising from lower gastrointestinal tract contribute to the development of systemic hypertension in diseases like inflammatory bowel disease.

## Author Contributions

ZM and CR: idea, writing, and editing. DO’L: idea and editing. All authors contributed to the article and approved the submitted version.

## Conflict of Interest

The authors declare that the research was conducted in the absence of any commercial or financial relationships that could be construed as a potential conflict of interest.

## Publisher’s Note

All claims expressed in this article are solely those of the authors and do not necessarily represent those of their affiliated organizations, or those of the publisher, the editors and the reviewers. Any product that may be evaluated in this article, or claim that may be made by its manufacturer, is not guaranteed or endorsed by the publisher.
